# Combination systemic therapies with immune checkpoint inhibitors in pancreatic cancer: overcoming resistance to single-agent checkpoint blockade

**DOI:** 10.1186/s40169-018-0210-9

**Published:** 2018-10-08

**Authors:** Jun Gong, Andrew Hendifar, Richard Tuli, Jeremy Chuang, May Cho, Vincent Chung, Daneng Li, Ravi Salgia

**Affiliations:** 1 Department of Gastrointestinal Malignancies, Cedars-Sinai Medical Center, 8700 Beverly Blvd, AC 1042C, Los Angeles, CA 90048 USA; 2 Department of Radiation Oncology, Cedars-Sinai Medical Center, 8700 Beverly Blvd, AC 1023, Los Angeles, CA 90048 USA; 3 Department of Internal Medicine, Harbor-UCLA Medical Center, 1000 W Carson St, Box 400, Torrance, CA 90509 USA; 4 Department of Internal Medicine, Division of Hematology and Oncology, UC Davis Comprehensive Cancer Center, 4501 X Street, Ste 3016, Sacramento, CA 95817 USA; 5 Department of Medical Oncology, City of Hope National Medical Center, 1500 E Duarte Rd, Bldg 51, Duarte, CA 91010 USA; 6 Medical Oncology and Experimental Therapeutics, City of Hope Comprehensive Cancer Center, Building 51, Room 101, 1500 E Duarte St, Duarte, CA 91010 USA

**Keywords:** Pancreatic cancer, Checkpoint inhibitor, Combination therapy, Immuno-oncology, Clinical trials

## Abstract

Immune checkpoint inhibitors have demonstrated broad single-agent antitumor activity and a favorable safety profile that render them attractive agents to combine with other systemic anticancer therapies. Pancreatic cancer has been fairly resistant to monotherapy blockade of programmed cell death protein 1 receptor, programmed death ligand 1, and cytotoxic T-lymphocyte associated protein 4. However, there is a growing body of preclinical evidence to support the rational combination of checkpoint inhibitors and various systemic therapies in pancreatic cancer. Furthermore, early clinical evidence has begun to support the feasibility and efficacy of checkpoint inhibitor-based combination therapy in advanced pancreatic cancer. Despite accumulating preclinical and clinical data, there remains several questions as to the optimal dosing and timing of administration of respective agents, toxicity of combination strategies, and mechanisms by which immune resistance to single-agent checkpoint blockade are overcome. Further development of biomarkers is also important in the advancement of combination systemic therapies incorporating checkpoint blockade in pancreatic cancer. Results from an impressive number of ongoing prospective clinical trials are eagerly anticipated and will seek to validate the viability of combination immuno-oncology strategies in pancreatic cancer.

## Introduction

Monoclonal antibodies targeting the programmed cell death protein 1 receptor (PD-1) and programmed death ligand 1 (PD-L1) are approved as cancer immunotherapy for a number of solid tumors and hematologic malignancies [1]. Early studies demonstrated expression of PD-L1 in human pancreatic cancer tissues associated with poor prognosis and evidence of antitumor activity with PD-1/PD-L1 blockade in pancreatic cancer mouse models in vivo [2–5]. However, pancreatic cancer has been fairly resistant to single-agent checkpoint blockade in the clinical setting as initial phase I trials enrolling advanced pancreatic cancer patients produced overall response rates (ORRs) of 0% with anti-PD-1 and anti-PD-L1 therapy [6–8]. Similarly, no objective responses were seen in locally advanced or metastatic pancreatic cancer patients treated with cytotoxic T-lymphocyte associated protein 4 (CTLA-4) inhibitor monotherapy [9]. These negative but important clinical studies underscored the primary or innate resistance of pancreatic cancer to checkpoint inhibitors rather than acquired resistance, which would otherwise be seen in those that initially respond but eventually develop resistance to these agents [10].

There is increasing evidence to suggest that both tumor cell-intrinsic and tumor cell-extrinsic factors contribute to the primary resistance of pancreatic cancer to checkpoint blockade. Immune active tumors that are sensitive to checkpoint inhibitors such as melanoma, lung squamous cell carcinoma, or lung adenocarcinoma are characterized by an abundance of CD8+ tumor-infiltrating lymphocytes (TILs), while pancreatic cancer (except those with defects in mismatch repair) represents an immune quiescent tumor characterized by lack of infiltration by effector T-cells that is otherwise critical in driving the antitumor response to checkpoint blockade [11]. This property of pancreatic adenocarcinoma (PDAC) has been attributed, in part, to the prominent PDAC desmoplastic stroma that may impede access to the tumor by TILs [12]. Additionally, PDAC is intrinsically a low mutational burden or low neoantigen-expressing tumor, which is important given that higher mutational load correlates to higher levels of neoantigens capable of inducing antitumor responses to immune checkpoint blockade [12].

Other tumor cell-intrinsic and tumor cell-extrinsic mechanisms of resistance to immune checkpoint inhibitors in pancreatic cancer include (1) the ability of pancreatic cancer cells to evade the host antitumor immune response (immunoediting) through expression of immune checkpoints such as PD-L1 and indoleamine-2,3-dioxygenase (IDO), secretion of granulocyte–macrophage colony-stimulating factor (GM-CSF) resulting in a myeloid cell-inflamed phenotype, and upregulation of regulatory T-cells (Tregs) and (2) induction of immune tolerance by direct interaction between cancer cells and tumor antigen-specific T-cells (immune privilege) through downregulation of antigen presenting major histocompatibility complex (MHC) molecules, expression of Fas ligand and decreased Fas receptor signaling, and expression of Foxp3 [13, 14].

Evidence is emerging to support combination systemic therapies on a backbone of immune checkpoint inhibition to overcome resistance to single-agent PD-1/PD-L1/CTLA-4 blockade in pancreatic cancer. PD-1/PD-L1 inhibitors, in particular, have shown broad single-agent activity across a spectrum of cancers with safety and tolerability profiles that render them attractive agents for combination with other anticancer therapies [1]. In this review, we highlight the current developmental landscape of combination regimens incorporating systemic therapies and checkpoint blockade in pancreatic cancer. In particular, we review the preclinical evidence supporting the rational combination and transition to prospective clinical trials that have thus far reported on the safety and efficacy of combination systemic therapies with checkpoint inhibitors in pancreatic cancer. We end with a discussion on future considerations that are important to optimizing the antitumor efficacy of these combinations in this lethal malignancy.

## Search criteria

A literature search up to April 17, 2018 using the keywords “pancreatic cancer,” “PD-1,” “PD-L1,” “checkpoint,” and “immunotherapy” was conducted in MEDLINE and generated a total of 1836 hits. Preclinical or prospective clinical studies investigating combination regimens involving PD-1, PD-L1, or CTLA-4 inhibitors and ≥ 1 systemic therapies in the treatment of pancreatic cancer were included. An additional manual search was performed to include preliminary results from relevant abstracts investigating combination therapies. Only studies published in English language were included. Using these criteria, the list of studies was narrowed to a total of 30 preclinical studies (22 fully published and 8 abstracts) and 14 prospective clinical studies (5 fully published and 9 abstracts) that were included in this review.

## Preclinical evidence

### Chemotherapy

Among the earliest preclinical studies demonstrating synergistic antitumor effects with combination checkpoint inhibition and chemotherapy in pancreatic cancer involved the administration of anti-PD-L1 therapy and gemcitabine in mice models in vivo without overt toxicity (Table 1). Gemcitabine with delayed anti-PD-L1 therapy (≥ 14 days after gemcitabine) and gemcitabine with simultaneous PD-L1 blockade showed enhanced tumor suppression compared to either modality alone; however, only simultaneous combination therapy resulted in complete responses (CRs) in treated mice [4]. In a transgenic mouse model of resectable PDAC, neoadjuvant PD-1 inhibition and gemcitabine significantly reduced local recurrence and improved survival compared to either modality alone, while promotion of natural killer (NK) cell activation with the addition of anti-CD96 antibody to adjuvant gemcitabine enhanced control of distant metastases [15]. This preclinical model therefore highlighted the potential of combination strategies with PD-1 blockade to target acquired resistance (preventing recurrence) in addition to the more commonly investigated goal of enhancing response to PD-1 inhibition (targeting primary resistance). Administration of cisplatin using nanoparticle carriers along with anti-PD-L1 therapy produced preliminary evidence of enhanced tumor suppression in an orthotopic mouse model of pancreatic cancer [16].

**Table 1:** Published preclinical evidence supporting combination regimens incorporating immune checkpoint inhibitors in pancreatic cancer

ICI	Non-ICI systemic therapy	Source	Refs.
Anti-PD-L1 mAb IP 0.3 mg 3× per week ×4 weeks	Gemcitabine IP 60 μg/g D1, 4, 7, 10, and 13 ×2 weeks	Mice PAN 02	[4]
Anti-PD-L1 mAb IP 160 μg every 48 h up to 6 days	CXCL12 inhibitor (AMD3100) osmotic pump at 30 mg/mL or 90 mg/mL up to 6 days	Mice KPC	[32]
CTLA-4 inhibitor IP 250 μg/dose or PD-1 inhibitor IP 200 μg/dose every 4–5 days	CSF1 neutralizing antibody IP every 4–5 days (1 mg for first dose then 0.5 mg) or CSF1R inhibitors 800 mg/kg in chow	Mice KRAS-INK	[17]
PD-1-CD28 fusion receptor-transduced OT-1 T-cells IV cocultured for 48 h at a ratio 10:1 T-cell/tumor cells	PD-1-CD28 fusion receptor-transduced OT-1 T-cells	Mice PANC 02-OVA	[39]
Anti-PD-1 mAb IP 100 μg or anti-PD-L1 mAb IP 100 μg on D3 after inoculation → every 2 weeks	Cyclophosphamide 100 mg/kg IP X1 on D3 after inoculation + GVAX SC in 3 limbs (0.1 mL) on D4, 7, 14, and 21	Mice PANC 02	[28]
Anti-PD-1 IP 200 μg D0, 3, 6, 9, 12, 15, 18, and 21 and/or anti-CTLA-4 IP 200 μg D0, 3, and 6 after enrollment	Gemcitabine + nab-paclitaxel IP 120 mg/kg D1 and CD40 agonistic antibody IP 100 μg D3	Mice KPC	[42]
Anti-PD-L1 mAb IP 10 mg/kg twice weekly on D7 or D14 after inoculation ×6 doses	CD40 agonistic antibody IP 3 mg/kg once weekly on D7 after inoculation ×4 doses	Mice PAN 02	[41]
Anti-CTLA-4 mAb IP 250 μg or anti-PD-1 mAb IP 200 μg every 4–5 days	FAK inhibitor (VS-4718) oral gavage 50 mg/kg twice daily and/or gemcitabine IV 25 mg/kg every 4–5 days	Mice KP, KPPC	[18]
Anti-PD-1 mAb IP 20 mg/kg every 3 to 4 days on D3 after inoculation	NaHCO_3_ oral drinking water 200 mM ad lib 3 days prior to inoculation	Mice PANC 02	[25]
Anti-PD-1 mAb IP 10 mg/kg twice weekly	CXCR2 SM oral 100 mg/kg twice daily	Mice KPC	[36]
Anti-PD-L1 mAb IP 200 μg twice weekly up to D21	Local RT 20 Gy on D0 and 15 Gy on D7 + 2 × 10^6^ MC57-SIY cells (vaccine) on D0 with 2 SC doses of boosted vaccine (10 μg SIY peptide and 20 μg poly I:C each) on D7 and D21	Mice PANC02-SIY	[30]
Anti-PD-L1 antibody 200 μg/mouse) every 2 days ×10 days	MLL1 inhibitor (verticillin A) 0.5 mg/kg body weight every 2 days ×10 days	Mice PANC02-H7	[19]
Anti-PD-1 mAb IP 200 μg every 2 days ×10 days	Ruxolitinib oral gavage 50 mg/kg starting on D5 after inoculation daily ×10 days	Mice PANC02-H7	[21]
Bispecific PD-L1 and CXCL12 trap IV 50 μg plasmid/mice every 2 days ×4 doses starting on D13 after inoculation	Bispecific PD-L1 and CXCL12 trap	Mice KPC-RFP/luc	[33]
Anti-PD-1 mAb IP 200 μg every 3 days as needed ×18 days starting on D7 after inoculation	MEK inhibitor IP 1 mg/kg daily ×18 days starting on D7 after inoculation	Mice 65 671	[22]
PD-1 or PD-L1 inhibitor IP (dose not specified) once weekly 1 week after inoculation	IL-18 inhibitor (IL-18BP) IP (dose not specified) once weekly 1 week after inoculation	Mice PANC 02-luc	[35]
Anti-PD-L1 mAb IP 200 μg/mouse 3× per week ×2 weeks	Anti-IL-6R mAb IP 200 μg/mouse 3× per week ×2 weeks	Mice KPC-BRCA 2, MT5, PANC 02, or KPC-luc	[34]
Anti-PD-L1 mAb IP 200 μg every 1 and 3 days after PolyICLC injection	mAb-AR20.5 IP 50 μg D7, 17, 27 and 37 + PolyICLC IP 50 μg D8, 13, 18, 23, 28, 33, 38, and 43	Mice PANC02.MUC1, KPC.MUC1	[43]
Anti-PD-1 mAb IP 10 mg/kg 2× per week on D10 after inoculation	Anti-BAG3 antibody IP 20 mg/kg 3× per week on D10 after inoculation	Mice mt4-2D	[20]
A12-IFNγ (IP 5 μg/mouse daily ×18 days) or B3-IL2 (IP 1 μg/mouse daily ×18 days) fusion compounds	A12-IFNγ: single-domain antibodies against PD-L1 fused with IFNγ B3-IL2: single-domain antibodies against PD-L1 fused with IL-2	Mice PANC 02, KPC, KPC organoids	[38]
Anti-PD-1 mAb IP 125 μg ×3 doses or 200 μg every 3 days or anti-CTLA-4 mAb IP 200 μg every 3 days on D7 after inoculation	CCK-A receptor inhibitor (L364,718) ILP 4 mg/kg 3× per week or CCK-B receptor inhibitor (proglumide) oral 30 mg/kg daily on D7 after inoculation	Mice PANC 02, mT3-2D	[23]
Neoadjuvant anti-PD-1 mAb IP 150 μg every 3 days ×3 doses starting 20 days after electroporation	Neoadjuvant gemcitabine IP 100 mg/kg every 3 days ×3 doses (starting 20 days after electroporation) followed by surgical resection on D8 followed by adjuvant gemcitabine weekly ×5 doses or anti-CD96 mAb IP 250 μg twice weekly ×6 doses + gemcitabine weekly ×7 doses	Mice transgenic (KrasG12V, myrAkt2, and SB13 plasmids and Cre recombinase	[15]

*ICI* immune checkpoint inhibitor, *PD-L1* programmed death ligand 1, *mAB* monoclonal antibody, *IP* intraperitoneal, *D* day, *CXCL12* chemokine (C-X-C motif) ligand 12, *CTLA-4*, cytotoxic T-lymphocyte associated protein 4, *PD-1* programmed cell death protein 1 receptor, *CSF1/CSF1R* colony-stimulating factor 1/colony-stimulating factor 1 receptor, *OT-1* ovalbumine, *GVAX* allogeneic pancreatic tumor cells transfected with granulocyte–macrophage colony-stimulating factor (GM-CSF) gene, *SC* subcutaneous, *FAK* focal adhesion kinase, *IV* intravenous, *CXCR2* C-X-C chemokine receptor type 2, *RT* radiation therapy, *Gy* gray, *MLL1* mixed-lineage leukemia 1, *MEK* mitogen-activated protein kinase (MAPK) kinase; *IL-18* interleukin 18, *IL-6R* interleukin 6 receptor, *mAb-AR20.5* anti-MUC1, *PolyICLC* toll like receptor-3 ligand, *BAG3* Bcl-2-Associated athanoGene 3, *IFNγ* interferon-γ, *CCK* cholecystokinin

### Targeted therapies

Preclinical work has demonstrated that tumor-associated macrophages (TAMs) and monocytic and granulocytic myeloid-derived suppressor cells (MDSCs) contribute to the immunosuppressive tumor microenvironment (TME) of pancreatic cancer [17]. In PDAC mouse models, inhibition of colony-stimulating factor 1 (CSF1) or colony-stimulating factor 1 receptor (CSF1R) decreased TAMs and reprogrammed TAMs to promote antigen presentation and antitumor T cell activity, increased CD3+CD8+ cytotoxic T-lymphocytes (CTLs) and CD3+CD4+ effector T-cells, decreased CD4+Foxp3+ Tregs, improved the effector T-cell/Treg ratio, and upregulated PD-L1 and CTLA-4 on PDAC cells. Combination CSF1/CSF1R and PD-1 or CTLA-4 blockade synergistically restrained tumor progression, compared to controls.

Hyperactivity of focal adhesion kinase (FAK) has been shown to promote tumor protective fibrosis and an immunosuppressive TME in PDAC. Addition of a FAK inhibitor reversed resistance to chemotherapy (gemcitabine) and checkpoint inhibition in PDAC-bearing mice models (Table 1). Enhanced sensitivity to PD-1 inhibition occurred when given in combination with low-dose gemcitabine 25 mg/kg and was associated with increased CD8+ CTLs that penetrated into the stroma in close proximity with target CK19+ PDAC cells, decreased CD4+Foxp3+ Tregs, and improved T-effector/Treg ratios in the tumors, when compared to controls [18].

Targeting of either the H3K4 methylation-specific histone methyltransferase, mixed-lineage leukemia 1 (MLL1), with the epigenetic agent verticillin A, JAK/STAT pathway with ruxolitinib, mitogen-activated protein kinase (MAPK) pathway with a MAPK kinase (MEK inhibitor, cholecystokinin (CCK) receptor, DNA methyltransferase with decitabine, or Bcl-2 in combination with checkpoint blockade significantly enhanced tumor growth suppression, when compared to controls, in pancreatic cancer-carrying mouse models [19–24]. MLL1 normally catalyzes the trimethylation of H3K4 to activate immune inhibitory PD-L1 transcription in tumor cells [19]. The JAK/STAT pathway putatively upregulates PD-L1 expression and immunosuppressive cytokine production by tumor cells that altogether decrease effector T-cell function [21]. Myeloid cells have been shown to support immune evasion in PDAC by upregulating PD-L1 expression in a MAPK-dependent manner [22]. CCK has been implicated in increasing fibrosis and reducing the influx of TILs in pancreatic cancer [23]. Decitabine, a DNA hypomethylating agent, was shown to increase the amount of CD8+ TILs as monotherapy. This prompted the addition of PD-1 antibody following decitabine treatment that showed greater suppression of tumor growth compared to either agent alone in PDAC mice [24]. Lastly, blocking of Bcl-2-Associated athanoGene 3 (BAG3) has been shown to decrease the number of immunosuppressive TAMs in PDAC [20].

### Tumor microenvironment

Preclinical evidence posits that the acidic pH of the TME has immunosuppressive effects by inhibiting T-cell activation and abrogating interferon-γ (IFN-γ) and tumor necrosis factor alpha (TNF-α) secretion [25]. Buffer therapy with sodium bicarbonate in drinking water and anti-PD-1 therapy significantly diminished tumor growth in a PDAC mouse model compared to either therapy alone (Table 1). Given the concerns for translating such high doses of sodium bicarbonate in the clinical setting, the same group has preliminarily demonstrated the ability to increase sensitivity to anti-PD-1 monotherapy by combining the carbonic anhydrase IX (CAIX) inhibitor DH348 or lactate dehydrogenase A inhibitor FX11 with anti-PD-1 therapy in PDAC-carrying mice [26].

Hyaluronan has been shown to contribute to tumor promotion and depletion by PEGylated recombinant human hyaluronidase PH20 (PEGPH20) 24 h prior to anti-PD-1 or anti-PD-L1 therapy significantly suppressed tumor growth, when compared to either modality alone, in high hyaluronan-expressing pancreatic tumor mice models [27]. Gene expression of immunosuppressive markers such as interleukin 10 (IL-10) and Foxp3 was higher in hyaluronan-high tumors and suggested a relationship between hyaluronan level and immune suppression.

### Vaccines

Combining cyclophosphamide and a GM-CSF cell-based vaccine (GVAX) with anti-PD-1 or anti-PD-L1 antibodies in mouse models of pancreatic cancer showed significantly increased survival compared to anti-PD-1 monotherapy (Table 1) [28]. Notably, combination systemic therapy was associated with significantly increased IFNγ-producing CD8+ TILs in the metastatic PDAC TME, decreased Tregs, and decreased CTLA-4 expression on CD4+ and CD8+ T-cells when compared to controls. The same group later demonstrated preliminary antitumor efficacy with the combination of Annexin A2-specific Listeria monocytogenes vaccine and anti-PD-1 therapy in a PDAC mouse model [29]. The pairing of checkpoint blockade to local radiation therapy (RT) and a SIY antigen vaccine enhanced tumor regression in otherwise immune quiescent pancreatic cancer mouse models (Table 1) [30].

### Cytokines and chemokines

Production of chemokines such as CCL2, CXCL12, and CXCR4 contribute to the immunosuppressive PDAC TME by facilitating T-cell trapping in the stroma and effector T-cell exclusion [31]. Regulation of cytokines such as interferons and TNF-α also contribute to the immunosuppressive PDAC TME through upregulation of PD-L1 [31]. Targeting the chemokine (C-X-C motif) ligand 12 (CXCL12) through the chemokine (C-X-C motif) receptor 4 (CXCR4) inhibitor, AMD3100, in PDAC-bearing mice with anti-PD-L1 therapy significantly reduced tumor volume by 48 h, when compared to controls, with no further decreases in tumor volume over the following 4 days [32]. Interestingly, CTLA-4 blockade did not augment the antitumor effect of AMD3100. A subsequent study established the efficacy of a bispecific PD-L1 and CXCL12 fusion protein or trap with > 1000× higher affinity for mouse PD-L1 than that between endogenous PD-1 and PD-L1 [33].

In preclinical PDAC mouse models, combined targeting of PD-L1 and IL-6 correlated with increased intratumoral effector T-cells and increased T-cells with a Th1 phenotype, while inhibiting pancreatic cancer growth compared to either modality alone [34]. Targeting of IL-18, CCR2, or CXCR2 in combination with PD-1/PD-L1 blockade has shown preclinical anticancer efficacy as well (Table 1) [35–37]. Delivery of an antitumor cytokine payload demonstrated feasibility in PDAC mice treated with single-domain antibodies against PD-L1 fused with IFNγ or IL-2 in vivo; reduced tumor burden seen from targeting IFNγ was associated with decreased numbers of CD11b^+^ cells and transition of intratumoral macrophages towards an M1-like phenotype [38].

### Adoptive T-cell therapy

In initial investigations of fusion receptor constructs comprised of PD-1 and the costimulatory protein CD28 transduced into transgenic murine CD8+ T-cells specific for ovalbumin (OT-1), complete tumor regressions in PDAC-carrying mice were observed with 300-fold increases in IL-2 and IFN-γ production and increased T-cell proliferation [39]. Reimplanted tumors were rejected in 9/11 treated mice vs. 0/6 naïve mice, which was indicative of a memory response. The same group has recently presented preliminary findings of synergistic T-cell-induced tumor cell cytotoxicity in mouse pancreatic cancer cell lines cocultured with OVA-specific CD4+ and CD8+ T-cells transduced with a PD-1-CD28 fusion receptor [40].

### Immune costimulatory proteins and immunostimulants

In studies demonstrating resistance to single-agent checkpoint blockade in PDAC-bearing mice, addition of CD40 (antigen presenting cell costimulatory protein) agonistic antibody and/or chemotherapy reversed refractoriness to checkpoint blockade by priming T-cell responses (Table 1) [41, 42]. Combination therapy significantly improved survival (targeting primary resistance) and conferred immunologic memory as demonstrated by curative protection from multiple tumor rechallenges, when compared to controls (targeting acquired resistance). Triple therapy with mAb-AR20.5 (anti-MUC1), anti-PD-L1 therapy, and PolyICLC (immunostimulant) cured 50% of mice subcutaneously injected with PDAC cells by 70 days and retained immunologic memory as evidenced by tumor antigen-specific rejection of tumors reimplanted in treated mice but not in control mice [43]. Preliminary antitumor efficacy has also been shown with triple therapy involving the immunomodulatory antifungal ciclopirox olamine, anti-PD-1, and anti-CTLA-4 therapy in PDAC-carrying mice [44].

## Prospective clinical trials

### Vaccines

In an open-label phase Ib trial, patients with previously gemcitabine-treated, advanced PDAC were randomized to receive ipilimumab (arm 1) or ipilimumab with GVAX (arm 2) and demonstrated grade 3–4 immune-related adverse events (AEs) in 20% of patients in both arms (colitis, Guillain-Barre syndrome (GBS), and nephritis in arm 1 and colitis, rash, and pneumonitis in arm 2) that resolved to grade 1 with steroids (except for the cases of nephritis and GBS) [45]. There was a trend towards significant improvement in overall survival (OS) in favor of the combination arm despite the small sample size of 30 in this study (Table 2).

**Table 2:** Prospective clinical trials supporting combination regimens incorporating systemic therapies and immune checkpoint inhibitors in pancreatic cancer

Study design, setting	Treatment arms	Outcomes	Refs.
Phase Ib, previously gemcitabine-treated	Ipilimumab IV 10 mg/kg (n = 15) vs. ipilimumab + GVAX vaccine intradermal injection ×4 (n = 15) on weeks 1, 4, 7, and 10 → maintenance every 12 weeks if response or SD	Median OS 3.6 mos (95% CI 2.5–9.2) vs. 5.7 mos (95% CI 4.3–14.7, HR 0.51, 95% CI 0.23–1.08, p = 0.072) SD 2/15 (13.3%) vs. SD 3/15 (20%)	[45]
Phase Ib, 1st-line	Tremelimumab IV 6–15 mg/kg on D1 every 84-day cycles + G 1000 mg/m^2^ weekly ×3 weeks every 4-week cycles (n = 34)	Median OS 7.4 mos (95% CI 5.8–9.4) PR 2/19 (10.5%) at MTD of 15 mg/kg trememlimumab	[48]
Phase Ib, gemcitabine-naïve	MTD: Ipilimumab IV 3 mg/kg every 3 weeks ×4 doses + G 1000 mg/m^2^ weekly ×3 weeks every 4-week cycles (n = 16) → maintenance ipilimumab every 12 weeks and G weekly ×3 weeks every 4-week cycles	PR 2/16 (12.5%) Median PFS 2.5 mos (95% CI 0.8–4.8) Median OS 8.5 mos (95% CI 2.2–10.3)	[51]
Pilot study, NR	Monocyte derived dendritic cell vaccine (dose NR) + nivolumab IV 1–2 mg/kg 1 day before vaccine (n = 7)	2 PRs with OS after onset of therapy of 13 and 5 mos	[47]
Phase Ib, treatment-refractory	BGB-A317 (PD-1 inhibitor) IV 2 mg/kg or 200 mg every 3 weeks + BGB-290 (PARP 1/2 inhibitor) oral 20–60 mg twice daily (n = 38, advanced solid tumors)	7 PRs (1 pancreatic cancer) and 6 SD for > 6 mos (2 pts with pancreatic cancer who received BGB-A317 + BGB-290 for 189 and 281 days) Most common treatment-related AE was fatigue (10.5%) Immune-related AEs: Grade 3 hypophysitis (n = 1), grade 3–4 autoimmune hepatitis (n = 2), and grade 2 transaminitis (n = 1)	[55]
Phase Ib, 1st- and 2nd-line	Treatment-naïve: Pembrolizumab IV 2 mg/kg D1 + G 1000 mg/m^2^ + N 125 mg/m^2^ D1 and 8 every 21-day cycles (n = 11) Pretreated: Pembrolizumab + G 800 mg/m^2^ + N 100 mg/m^2^ D1 and 8 every 21-day cycles (n = 4)	Treatment-naïve: PR 3/11 (27.2%), median PFS 9.1 mos (95% CI 4.9–15.3), median OS 15 mos (95% CI 6.8–22.6) Pretreated: SD 2/4 (50%, closed due to futility)	[50]
Phase I, 1st- and 2nd-line	Arm A: Nivolumab IV 3 mg/kg D1 and 15 + N 125 mg/m^2^ D1, 8, and 15 every 28-day cycles (previously treated with 1 line of chemotherapy, n = 11) Arm B: Same as arm A with G 1000 mg/m^2^ weekly ×3 weeks every 4-week cycles (treatment-naïve, n = 6)	Arm A: PR 2/9 (22.2%) Arm B: PR 3/6 (50%)	[52]
Phase I, neoadjuvant	Arm A: Pembrolizumab IV 200 mg D1, 22, and 43 + X 825 mg/m^2^ twice daily (on days of RT only) + 50.4 Gy ×28 fractions ×28 days (n = 14) Arm B: X + RT only (n = 8)	Arm A: 10/14 resection (71.4%) with grade 3 AEs of diarrhea (n = 2), lymphopenia (n = 4), and elevated alkaline phosphatase (n = 1) Arm B: 4/14 resection (28.6%) with 1 grade 3 AE of lymphopenia	[53]
Phase I, treatment-refractory	Epacadostat oral 25 mg or 100 mg twice daily ± pembrolizumab IV 200 mg every 3 weeks (n = 15, 1 pancreatic cancer)	PR in pancreatic cancer pt who remains on therapy at 21 weeks	[56]
Phase I, treatment-refractory	M7824 (avelumab fused to the extracellular domain of TGFβ receptor II) IV 1, 3, 10, or 20 mg/kg every 2 weeks (n = 5)	MTD not reached with 1 each (n = 19) of grade ≥ 3 anemia, colitis, gastroparesis, hypokalemia, elevated lipase, and skin infection PR 1/5 (20%) SD 3/5 (60%)	[58]
Phase II, 2nd-line	Durvalumab IV 1.5 g IV every 4 weeks (n = 33) or durvalumab IV 1.5 g + tremelimumab IV 75 mg every 4 weeks ×4 doses → durvalumab IV 1.5 g every 4 weeks up to 12 mos (n = 32)	Monotherapy: 2 unconfirmed PRs (6.1%), DCR 6.1%, median PFS 1.5 mos, median OS 3.6 mos Combination: 1 PR (3.1%) > 12 mos, DCR 9.4%, median PFS 1.5 mos, and median OS 3.1 mos	[57]
Phase II, 1st-line	G 1000 mg/m^2^ D1, 8, and 15 + N 125 mg/m^2^ D1, 8, and 15 + durvalumab IV 1500 mg D1 + tremelimumab IV 75 mg D1 every 28-day cycles (n = 11)	PR 8/11 (73%, median duration 7.4 mos) DCR 100% Median PFS 7.9 mos (95% CI 3.5–9.2)	[54]

*IV* intravenous, *GVAX* allogeneic pancreatic tumor cells transfected with granulocyte–macrophage colony-stimulating factor (GM-CSF) gene, *SD* stable disease, *OS* overall survival, *CI* confidence interval, *HR* hazard ratio, *PR* partial response, *D* day, *MTD* maximum-tolerated dose, *PFS* progression-free survival, *NR* not reported, *PD-1* programmed cell death protein 1 receptor, *PARP* poly (ADP-ribose) polymerase, *G* gemcitabine, *N* nab-paclitaxel, *X* capecitabine, *RT* radiation therapy, *Gy* gray, *AE* adverse event, *TGFβ* transforming growth factor beta, *DCR* disease control rate

A German group isolated antigen-primed monocyte derived dendritic cells (DCs) from 44 patients with stage IV pancreatic cancer who failed first-line chemotherapy and demonstrated a median OS of 8 months with DC vaccine alone; however combination DC vaccine and PD-L1 blockade was able to induce secondary stabilization of disease of 4–8 months in 5/10 patients who failed to respond to previous DC therapy [46]. Preliminary results including 2 partial responses (PRs) were observed from a pilot study by the same group investigating lower dose nivolumab in combination with DC vaccine therapy in 7 patients with stage IV pancreatic cancer (Table 2) [47].

### Chemotherapy

The CTLA-4 inhibitor tremelimumab in combination with weekly gemcitabine demonstrated preliminary efficacy and tolerability with the most common grade 3–4 toxicities being asthenia (11.8%) and nausea (8.8%) in a phase Ib trial enrolling treatment-naïve metastatic pancreatic cancer patients [48]. A dose-finding, multi-arm phase Ib trial enrolled patients with advanced solid tumors to 6 different treatment arms; in the metastatic PDAC cohort, a final 17 patients with treatment-naïve or previously-treated disease received combination pembrolizumab, gemcitabine, and nab-paclitaxel with responses observed only in those who were previously untreated (Table 2) [49, 50]. Notably, gemcitabine and nab-paclitaxel were reduced to 800 mg/m^2^ and 100 mg/m^2^ in the pretreated cohort, which was ultimately closed due to futility [50]. Immune-related AEs (all grades) were seen in 47.1%. The most common grade 3–4 AEs were neutropenia (46.7%) and thrombocytopenia (20%) in the treatment-naïve cohort. The combination of ipilimumab and gemcitabine was well-tolerated but produced an ORR of 12.5% and lower than that of gemcitabine alone, historically [51].

Alternatively, the feasibility and preliminary efficacy of nivolumab + nab-paclitaxel (arm A) and nivolumab + gemcitabine + nab-paclitaxel (arm B) has been demonstrated in locally advanced or metastatic PDAC where the most common grade 3–4 AEs in arm A were pulmonary embolism, neutropenia, and anemia in 2/11 patients (18%) and anemia in 2/6 (33%) in arm B [52]. Neoadjuvant pembrolizumab with concurrent capecitabine and RT was relatively tolerated in resectable or borderline resectable PDAC with no grade 4 toxicities reported and no major surgical complications reported within 30 days post-surgery (Table 2) [53].

Most recently, preliminary results from a phase II trial highlighted the most promising efficacy findings to date with combination chemotherapy and checkpoint blockade in pancreatic cancer (Table 2) [54]. As part of a safety run-in for this trial, 11 treatment-naïve metastatic pancreatic cancer patients received combination gemcitabine + nab-paclitaxel + durvalumab + tremelimumab with the most common grade ≥ 3 AEs being hypoalbuminemia (45%), abnormal lipase (45%), anemia (36%), fatigue (27%), abnormal white blood cells (27%), and hyponatremia (27%). There was 1 patient (9.1%) who experienced grade 3 colitis.

### Targeted therapies

Preliminary findings from a phase Ib dose-finding trial of a PD-1 inhibitor plus PARP inhibitor in patients with treatment-refractory advanced solid tumors and demonstrated the feasibility of this pairing (Table 2) [55]. Furthermore, a PR and prolonged stable disease (SD) of up to 281 days were observed in pancreatic cancer patients treated with this combination.

### Other immune checkpoint inhibitors

Preliminary results from a phase I Japanese trial investigating the oral IDO inhibitor epacadostat and pembrolizumab showed a PR thus far in1 pancreatic cancer patient out of 15 patients with treatment-refractory advanced solid tumors (Table 2) [56]. In the overall cohort, there was 1 dose-limiting toxicity (DLT) of grade 4 rhabdomyolysis in the epacadostat 100 mg + pembrolizumab arm and 12 patients experienced all-grade AEs (80.0%) while 2 patients (13.3%) had grade 3 liver disorder and grade 4 rhabdomyolysis (1 each) with the combination.

A recent phase II trial reported preliminary findings of modest efficacy with durvalumab and tremelimumab in the second-line treatment of metastatic pancreatic cancer (Table 2) [57]. There were more grade ≥ 3 treatment-related AEs with combination durvalumab and tremelimumab (22%) than single-agent durvalumab (6%). Grade ≥ 3 treatment-related AEs seen in the combination arm included diarrhea (9.4%) and fatigue (6.3%), while ascites (3.1%), hepatitis (3.1%), and increased lipase (3.1%) were among those observed in the monotherapy arm. Discontinuation of therapy occurred in 9.4% and 3.1% of patients in the combination and monotherapy arms, respectively.

### Fusion proteins

A pilot phase I study demonstrated a PR in 1 patient with mismatch repair (MMR) deficiency out of 5 patients with pretreated advanced pancreatic cancer using a bifunctional fusion protein of anti-PD-L1 antibody fused to the extracellular domain of TGFβ receptor II (TGFβ trap) [58]. A maximum-tolerated dose (MTD) was not reached at the highest dose level of 20 mg/kg every 2 weeks (Table 2).

## Discussion

### Mechanisms of immune resistance and rational combination strategies

The mechanisms underlying the resistance of pancreatic cancer to the antitumor immune response have been extensively reviewed and are largely attributed to perturbations in immune surveillance, the process of immunoediting, and immune privilege [13, 59–61]. Although pancreatic cancer has been relatively resistant to single-agent checkpoint blockade when compared to more immune sensitive tumors, strategies for overcoming primary resistance to immunotherapy in pancreatic cancer are readily available from investigations in other solid tumor types focused on improving the antitumor efficacy of checkpoint blockade through combining various therapeutic modalities on a checkpoint inhibitor backbone [62–64]. Attractive agents to combine with checkpoint inhibitors should, in principle, directly stimulate CTLs, inhibit tumor-induced immunosuppressive factors, inhibit Tregs, and/or activate NK cell activity ideally through nonredundant pathways [63]. Here, for example, immunochemotherapy has been widely established across several tumor types where: (1) gemcitabine can increase class I human leukocyte antigen (HLA) expression, tumor antigen cross-presentation, and selectively eliminate MDSCs, (2) docetaxel can decrease immunosuppressive MDSCs, (3) paclitaxel can stimulate antigen-presenting cells and improve cancer cell permeability to granzyme B, (4) irinotecan can decrease Tregs and MDSCs, and (5) doxorubicin can promote immunogenic cell death, increase cancer cell permeability to granzyme B, and enhance antigen presentation by dendritic cells [49]. It has been shown, however, that vinorelbine may result in a bystander effect or the inadvertent death of neighboring immune cells that may interfere with the ability to mount an antitumor immune response [65].

Targeting the TME to attenuate immunosuppression, induce immunogenic tumor cell death, enhance antigen presentation, and/or prolong survival of immune-effector cells represents another approach to boost the anticancer immune response given the well-established immunosuppressive features of the PDAC TME [66]. Vitamin D priming may also serve as a potential adjunct to PDAC therapy given that the vitamin D receptor is expressed in the stroma of human pancreatic tumors and is involved in stromal remodeling and increased intratumoral drug delivery that may result in tumor volume reduction in combination strategies [67]. Furthermore, although checkpoint blockade alone is effective in removing immune suppression, it potentially does not provide a sustaining means for immune activation; strategies on priming antitumor immune-effector cells or rescuing dysfunctional immune-effector cells are also becoming increasingly recognized concepts to further improve the efficacy of checkpoint inhibitors in pancreatic cancer [11, 62, 68].

**Fig. 1:**
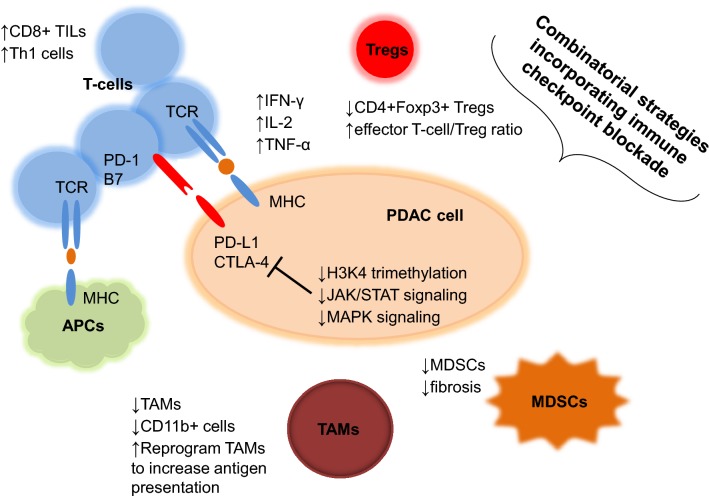
Mechanisms of immune resistance to checkpoint blockade in pancreatic cancer. Preclinical evidence supports that combinatorial strategies incorporating checkpoint inhibitors can attenuate primary and acquired resistance to checkpoint blockade through multiple tumor cell-intrinsic and tumor cell-extrinsic mechanisms. For example, targeting of H3K4 trimethylation, JAK/STAT signaling, and mitogen-activated protein kinase (MAPK) signaling mitigates tumor cell-intrinsic upregulation of PD-L1 expression. Combination regimens with checkpoint blockade can also target tumor cell-extrinsic mechanisms of immune resistance by decreasing tumor-associated macrophages (TAMs) or reprogramming TAMs to increase antigen presentation and antitumor T-cell activity. *MDSCs* myeloid-derived suppressor cells, *APCs* antigen-presenting cells, *MHC* major histocompatibility complex, *TCR* T-cell antigen receptor, *PD-1* programmed cell death protein 1 receptor, *B7* B7 family of ligands, *TILs* tumor-infiltrating lymphocytes, *PD-L1* programmed death ligand 1, *CTLA-4* cytotoxic T-lymphocyte associated protein 4, *IFNγ* interferon-γ, *IL-2* interleukin 2, *TNF-α* tumor necrosis factor alpha, *Tregs* regulatory T-cells

Indeed, a growing body of preclinical evidence supports the incorporation of checkpoint inhibitors in combination strategies with systemic therapies that address several pathways contributing to the immune resistance of pancreatic cancer (Fig. 1). For example, combination regimens incorporating checkpoint blockade have been shown to: (1) decrease TAMs and reprogram TAMs to promote antigen presentation and antitumor T-cell activity, (2) increase CD8+ TILs, decrease CD4+Foxp3+ Tregs, and improve effector T-cell/Treg ratios in tumors often associated with increased IL-2 and IFN-γ production and a Th1 phenotype, (3) upregulate PD-L1 and CTLA-4 on PDAC cells, (4) decrease numbers of CD11b^+^ cells with transition of intratumoral macrophages towards an M1-like phenotype, and (5) overcome the fibrotic and immunosuppressive TME of PDAC, while altogether producing enhanced antitumor activity when compared to controls [17, 18, 28, 34, 38, 39]. Notably, these studies fall into the same category as the overwhelming number of preclinical and clinical studies to date that have investigated the ability of combination regimens incorporating to PD-1/PD-L1/CTLA-4 inhibitors to target primary resistance or improve response to checkpoint blockade in pancreatic cancer (Tables 1 and 2). There is a small but relevant number of studies that have highlighted the potential for combination strategies with checkpoint inhibitors to target acquired resistance or prevent recurrence through the ability to induce immunologic memory to subsequent tumor rechallenge and reverse resistance to single-agent checkpoint blockade across several preclinical PDAC mouse models [39, 41–43].

### Candidates for combinations with immune checkpoint inhibitors

Based on the above rationale, a roadmap for partnering of therapeutic modalities with PD-1, PD-L1, and CTLA-4 inhibitors can be broadly conceptualized to strategies that can: (1) convert non-T-cell inflamed or immunologically “cold” tumors to T-cell inflamed or immunologically “hot” tumors and (2) enhance or rescue responses achieved with single-agent checkpoint blockade [69]. For the first approach that is essentially targeting primary resistance, evidence exists for vaccines, oncolytic viruses, immune cell co-stimulatory agonists (CD137 (4-1BB), CD134 (OX40), glucocorticoid-induced TNF receptor (GITR), and CD40), adoptive T-cell therapies and chimeric antigen receptor (CAR) T-cells, chemotherapy, RT, and targeted therapies as candidate agents for combination in this category [69]. The combination of RT and checkpoint blockade, in particular, is being explored extensively in clinical trials across numerous tumor types given the ability of RT to prime antitumor T-cell responses; the development of this combination as cancer therapy has been extensively reviewed elsewhere [70]. For the second approach that can target acquired resistance, inhibitors of other immunosuppressive molecules or immune checkpoints such as IDO, TGFβ inhibitors, angiogenesis inhibitors, and Treg depletion represent potential candidates for combination with anti-PD-1/PD-L1/CTLA-4 antibodies [69].

### Translation into clinical settings: dosing, timing, toxicities, and treatment setting

To date, only phase I-II trials have reported out with various combination regimens incorporating PD-1, PD-L1, and CTLA-4 inhibitors in PDAC patients and overall, they have shown modest efficacy that certainly represent improvements over the dismal response rates seen in clinical trials of single-agent checkpoint blockade in advanced PDAC (Table 2) [6–9]. However, combination therapies with checkpoint inhibitors in pancreatic cancer are still in early phases of clinical development and there are no large, randomized phase III trials for comparison to current treatment standards. Nevertheless, there are several points to consider from current clinical trials investigating combination regimens integrating checkpoint blockade in pancreatic cancer that will likely inform future studies in this area.

In an early phase I trial investigating the combination of first-line tremelimumab and gemcitabine in metastatic pancreatic cancer, the pharmacokinetics (PKs) of gemcitabine was similar in the absence or presence of tremelimumab at the highest dose level of 15 mg/kg [48]. The MTD was full-dose gemcitabine 1000 mg/m^2^ (weekly for 3 weeks with 1 week off) and tremelimumab 15 mg/kg on day 1 every 84-day cycles. The most common grade 3–4 treatment-related AE at the MTD was neutropenia (18%) and toxicity associated with combination therapy was essentially similar to that of tremelimumab monotherapy. Several phase I-II trials have since demonstrated the feasibility of adding standard-dose checkpoint inhibitors to full-dose gemcitabine and/or nab-paclitaxel in the first- and second-line treatment settings in advanced pancreatic cancer (Table 2) [49–52, 54]. Immune-related AEs have ranged from 5.9% to 47.1% (all grades) with combination chemotherapy and anti-PD-1 or anti-CTLA-4 therapy [48, 50]. Premedication with intravenous dexamethasone 12 mg on the day of chemotherapy infusion was shown to decrease grade 3–4 AEs (2.2 events/patient) compared to those without premedication (1.1 events/patient) [50].

Efficacy to combination chemotherapy and checkpoint blockade appears to be better in the treatment-naïve setting compared to the second-line setting in advanced PDAC, which posits that combination regimens with checkpoint inhibitors may be better served when introduced earlier in the treatment sequencing of metastatic PDAC regimens [50, 52]. There appears to be increased toxicities with greater numbers of agents or therapeutic modalities included with checkpoint inhibition [52–54]. Recently, the most promising efficacy seen to date occurred in a phase II trial investigating the quadruplet regimen of gemcitabine + nab-paclitaxel + durvalumab + tremelimumab in treatment-naïve metastatic PDAC with the most common grade ≥ 3 AEs being hypoalbuminemia (45%), abnormal lipase (45%), anemia (36%), fatigue (27%), abnormal white blood cells (27%), and hyponatremia (27%) [54]. In phase I-II trials to date, combination chemotherapy with PD-1/PD-L1/CTLA-4 inhibitors have employed concurrent administration of chemotherapy and checkpoint blockade [48–52, 54]. In the neoadjuvant treatment of borderline resectable PDAC, chemoradiation was also initiated concurrently with pembrolizumab in a phase Ib/II trial [53]. There is preclinical data to support simultaneous administration given that concurrent chemotherapy and PD-L1 blockade resulted in CRs that were not seen with delayed anti-PD-L1 therapy (≥ 14 days after gemcitabine) [4]. Notably, in a phase I dose-escalation trial of first-line gemcitabine and tremelimumab in metastatic pancreatic cancer, addition of tremelimumab 1 month after initiation with gemcitabine showed 0 grade 3–4 AEs of diarrhea compared to 2 grade 3–4 AEs of diarrhea in the concurrent therapy group though there was no significant difference in events of diarrhea over all cycles between arms (45% vs. 27%, respectively, p = 0.505) [48].

Phase I-II trials of dual checkpoint blockade or combination immunotherapy with checkpoint blockade in previously-treated advanced PDAC have similarly shown toxicity profiles typical of the respective agents [45, 57]. Specifically, the safety profile of combination GVAX + ipilimumab in PDAC patients was similar to that of ipilimumab alone in melanoma patients [45]. Combination durvalumab and tremelimumab, however, had higher grade ≥ 3 AEs (22%) than durvalumab alone (6%) leading to more treatment discontinuations (9.4% vs. 3.1%) [57]. Although limited by a small sample size of 5 patients with pretreated advanced pancreatic cancer, treatment with a bifunctional anti-PD-L1 and TGFβ receptor II fusion protein was efficacious and well-tolerated with a MTD not reached at the highest dose, altogether highlighting the feasibility of multitargeted fusion constructs involving checkpoint blockade [58].

A phase Ib investigating ipilimumab vs. ipilimumab + GVAX vaccine in gemcitabine-treated locally advanced or metastatic PDAC was among the first to demonstrate an interesting immuno-oncology concept in pancreatic cancer: only in the combination immunotherapy arm were delayed SD and radiographic responses associated with declines in tumor markers seen, and 2 of such cases had localized disease while 1 patient had lung-only metastases [45]. Furthermore, most patients demonstrating a response required ≥ 12 weeks of therapy. The authors proposed that immunotherapy should be initiated earlier in the treatment course, i.e., locally advanced or resected disease, to allow more time to induce immune responses as well as produce less immune tolerance given a smaller disease burden. Selecting PDAC patients in this manner will also allow patients who have better reserve more time to recover from immune-related AEs and be eligible for retreatment. Indeed, this concept of earlier integration of checkpoint blockade and immunotherapy in patients with likely lower disease burden has shown promising efficacy in the neoadjuvant, consolidative (after definitive therapy), and adjuvant settings in other tumor types [71–75]. There are several ongoing clinical trials investigating combination systemic therapies with checkpoint blockade in the perioperative treatment of pancreatic cancer to see if such an immuno-oncology approach is beneficial in the non-metastatic setting (Table 3).

**Table 3:** Ongoing clinical trials investigating combination regimens incorporating systemic therapies and immune checkpoint inhibitors in pancreatic cancer

Study/phase	n (patients needed)	Setting	Regimen	Primary outcome
NCT02648282/phase II	54	Locally advanced	CY + GVAX + PD-1 + SBRT	Distant metastasis free survival
NCT02451982/phase I/II	50	Neoadjuvant/adjuvant	CY/GVAX vs. CY/GVAX + nivolumab	Median IL17A expression
NCT03190265/phase II	63	Metastatic	Nivolumab/ipilimumab/CRS-207 + CY/GVAX vs. Nivolumab/ipilimumab/CRS-207	ORR
NCT03168139/phase I/II	20	Metastatic	Olaptesed pegol + pembrolizumab	Pharmacodynamics + safety
NCT03161379/phase II	50	Neoadjuvant	CY/GVAX + nivolumab + SBRT	Pathologic complete response
NCT03006302/phase II	70	Metastatic	Epacadostat/pembrolizumab/CRS-207 + CY/GVAX vs. Epacadostat/pembrolizumab/CRS-207	Recommended Dose of Epacadostat + 6 Month Survival
NCT03481920/phase I	24	Locally advanced/metastatic	Pegylated Hyaluronidase + avelumab	ORR + safety
NCT02734160/phase I	37	Metastatic	Galunisertib + durvalumab	DLT
NCT02983578/phase II	75	Locally advanced/metastatic	AZD9150 (antisense STAT3) + durvalumab	Disease Control Rate
NCT03451773/phase Ib/II	41	Locally advanced/metastatic	M7824 (TGF-beta + PD-L1 inhibitor) + gemcitabine	Safety and tolerability
NCT02403271/phase Ib/II	124	Locally advanced/metastatic	Ibrutinib + durvalumab	ORR + safety and tolerability
NCT01896869/phase II	92	Metastatic	Ipilimumab + vaccine vs. FOLFIRINOX	OS
NCT02451982/phase I/II	50	Neoadjuvant/adjuvant	CY (day 0) + GVAX (day 1 and 6–10 days after surgery ×4 + adjuvant CRT vs. CY (day 0) + GVAX (day 1 and 6–10 weeks after surgery ×4 + nivolumab (day 0 and 6–10 weeks after surgery)	Median IL17A expression
NCT02548169/phase I	20	Neoadjuvant	Arm A: Dendritic cell vaccine + standard of care chemotherapy Arm B: Dendritic cell vaccine + standard of care chemotherapy in metastatic disease	Safety and feasibility
NCT02243371/phase II	96	Metastatic	Arm A: CRS-207 + GVAX + nivolumab Arm B: CRS-207 + GVAX	OS
NCT02268825/phase I	39	Locally advanced/metastatic	Pembrolizumab + FOLFOX	Safety
NCT02303990/phase I	70	Locally advanced/metastatic	Pembrolizumab + RT	Adverse events
NCT02930902/phase Ib	30	Neoadjuvant	Pembrolizumab + paricalcitol vs. pembrolizumab + paricalcitol & standard chemo	Toxicity profile, Number of Tumor Infiltrating Lymphocytes
NCT03264404/phase II	31	Locally advanced/metastatic	Pembrolizumab + azacitadine	PFS
NCT02907099/phase II	15	Metastatic	BL-8040 + pembrolizumab	ORR
NCT02648282/phase II	54	Locally advanced	CY + GVAX + pembrolizumab + SBRT	Distant Metastasis Free Survival
NCT02546531/phase I	50	Locally advanced	Dose escalation and expansion: defactinib + pembrolizumab + gemcitabine	Recommended phase II dose
NCT02758587Phase I/II	59	Locally advanced	Defactinib + pembrolizumab	Adverse events
NCT03519308/phase I	20	Perioperative	nivolumab + nab-paclitaxel + gemcitabine + paricalcitol vs. nivolumab vs. nab-paclitaxel vs. gemcitabine	Adverse events
NCT03336216/phase II	160	Locally advanced/metastatic	Arm A: Gemcitabine/nab-paclitaxel or 5-fluorouracil/leucovorin/irinotecan Arm B: Cabiralizumab and nivolumab Arm C: cabiralizumab/nivolumab + gemcitabine/abraxane Arm D: cabiralizumab/nivolumab + oxaliplatin/5- fluorouracil/leucovorin	PFS
NCT03104439/phase II	80	MSI/MSS	Nivolumab + ipilimumab + RT	Disease control rate
NCT03214250/phase Ib/II	105	Metastatic	Arm A: Gemcitabine + nab-paclitaxel + nivolumab Arm B: Gemcitabine + nab-paclitaxel + APX005M (CD40 agonistic monoclonal antibody) Arm C: Gemcitabine + nab-paclitaxel + nivolumab + APX005 M	Adverse events, OS
NCT03404960/phase 1b/II	84	Locally advanced/metastatic	Niraparib + nivolumab	PFS
NCT03184870/phase I/II	260	Metastatic	Arm A: BMS-813160 + 5-fluorouracil (5-FU) + leucovorin + irinotecan Arm B: BMS-813160 + nab/paclitaxel + gemcitabine Arm C: BMS-813160 + nivolumab Arm D: BMS-813160	Adverse events, death, ORR, PFS
NCT03250273/phase II	54	Metastatic	Entinostat + nivolumab	ORR
NCT02754726/phase II	10	Metastatic	Nivolumab + paclitaxel + paricalcitol + cisplatin + gemcitabine	Complete response rate
NCT03373188/phase I	32	Neoadjuvant	Arm A: surgery only Arm B: VX15/2503 (anti-SEMA4D monoclonal antibody) + surgery Arm C: VX15/2503 + ipilimumab + surgery Arm D: VX15/2503 + nivolumab + surgery	Tumor CD8 + T cell infiltration between treatment groups
NCT03098550/phase I/II	120	Locally advanced/metastatic	Nivolumab + daratumumab	Tolerability
NCT02777710/phase I	58	Locally advanced/metastatic	Pexidartinib + durvalumab	Dose limiting toxicities, ORR
NCT02866383/phase II	80	Metastatic	Arm A: Nivolumab + RT Arm B: Nivolumab + ipilimumab + RT	Clinical benefit rate
NCT03098160/phase I	69	Locally advanced/metastatic	Evofosfamide + Ipilimumab	Recommended phase II dose
NCT02879318/phase II	180	Metastatic	Arm A: Gemcitabine + nab-paclitaxel Arm B: Gemcitabine + nab-paclitaxel + durvalumab + tremelimumab	OS
NCT02658214/phase Ib	42	Locally advanced/metastatic	Durvalumab + tremelimumab + nab-paclitaxel + gemcitabine	Adverse events, tumor assessment, laboratory findings

*PD-1* programmed cell death protein 1 receptor, *PD-L1* programmed death ligand 1, *CTLA-4* cytotoxic T-lymphocyte associated protein 4, *CY* cyclophosphamide, *ORR* objective response rate, *DLT* dose-limiting toxicities, *FOLFIRINOX* folinic acid, 5-fluorouracil, irinotecan, and oxaliplatin, *OS* overall survival, *GVAX* GVAX, allogeneic pancreatic tumor cells transfected with granulocyte-macrophage colony-stimulating factor (GM-CSF) gene, *SBRT* stereotactic body radiation therapy, *TGF* transforming growth factor, *FOLFOX* 5-fluorouracil, folinic acid, and oxaliplatin, *PFS* progression-free survival, *MSI/MSS* microsatellite instability/microsatellite stable, *RT* radiotherapy

### Biomarkers

Regardless of the treatment setting in the pancreatic cancer, biomarkers to guide the optimal selection of candidates for immune checkpoint blockade-based therapies are desperately needed for this malignancy. Several meta-analyses have corroborated the prognostic value of PD-L1 expression in PDAC patients, however, its utility as a predictive biomarker for checkpoint inhibitors has yet to be as validated as it has been in other tumor types [76, 77]. Tumors with mismatch repair deficiency (dMMR)/microsatellite instability (MSI) or high tumor mutation burden (TMB) have been shown to respond to checkpoint inhibitors though dMMR is relatively rare in PDAC (frequency of 0.8%) and pancreatic cancer has among the lowest TMB across tumor subtypes [78, 79]. However, investigations are ongoing and greater efforts are being undertaken to identify novel immuno-oncology biomarkers in pancreatic cancer. Recent correlation of cytolytic immune activity with mutational, structural, and neoepitope features in human PDAC samples has identified potential genomic signatures predictive of low cytolytic T-cell activity and expression signatures for multiple immune checkpoints other than PD-1/PD-L1 predictive of high immune cytolytic activity that altogether provide impetus for further investigation into therapeutic strategies that target multiple other immune checkpoints in pancreatic cancer [80]. An abundant microbiome within the pancreatic tumor that is distinct from that of the gut has interestingly been shown to promote immunosuppression that is characteristic of PDAC; targeting of this microbiome reverses tumor immune tolerance and enables efficacy for checkpoint inhibition-based therapy [81]. Continued advances in immuno-oncology biomarker development will ideally allow for selection of PDAC patients most likely to benefit from checkpoint blockade-based therapies based on a comprehensive immune profile—an individualized approach that remains a cornerstone to achieving precision immuno-oncology [82].

## Conclusion

Despite the dismal activity of single-agent checkpoint blockade in pancreatic cancer, PD-1, PD-L1, and CTLA-4 inhibitors have shown broad antitumor activity as single agents in other tumor types and a relatively tolerable toxicity profile rendering them attractive agents to combine with systemic therapy. Indeed, there is a growing body of early clinical evidence to suggest the feasibility and efficacy in combining checkpoint blockade with other forms of systemic therapy in pancreatic cancer. Although phase I-II data support the concurrent administration of standard-dose checkpoint blockade with full-dose systemic therapies including chemotherapy, there remains several questions on dosing, timing, toxicity, and patient selection for checkpoint inhibitor-based combination therapies in pancreatic cancer that warrant further prospective validation. Results from ongoing clinical trials investigating combination strategies with checkpoint blockade in pancreatic cancer are eagerly awaited and will hopefully provide answers to many looming questions in this arena (Table 3).

## Abbreviations

AEs: adverse events
CAIX: carbonic anhydrase IX
CAR: chimeric antigen receptor
CCK: cholecystokinin
CSF1: colony-stimulating factor 1
CSF1R: colony-stimulating factor 1 receptor
CR: complete response
CTLA-4: cytotoxic T-lymphocyte associated protein 4
CTLs: cytotoxic T-lymphocytes
CXCL12: chemokine (C-X-C motif) ligand 12
CXCR4: chemokine (C-X-C motif) receptor 4
DCs: dendritic cells
DLT: dose-limiting toxicity
dMMR: mismatch repair deficiency
FAK: focal adhesion
IDO: indoleamine-2,3-dioxygenase
IFN-γ: interferon-γ
IL: interleukin
GBS: Guillain–Barre syndrome
GITR: glucocorticoid-induced TNF receptor
GM-CSF: granulocyte–macrophage colony-stimulating factor
GVAX: GM-CSF cell-based vaccine
HLA: human leukocyte antigen
MAPK: mitogen-activated protein kinase
MDSCs: monocytic and granulocytic myeloid-derived suppressor cells
MEK: MAPK kinase
MHC: major histocompatibility complex
MLL1: mixed-lineage leukemia 1
MMR: mismatch repair
MSI: microsatellite instability
MTD: maximum-tolerated dose
TGFβ: transforming growth factor beta
Anti-MUC1: mAb-AR20.5
NK: natural killer
ORR: overall response rate
OS: overall survival
OT-1: ovalbumin
PD-1: programmed cell death protein 1 receptor
PD-L1: programmed death ligand 1
PDAC: pancreatic adenocarcinoma
PEGPH20: PEGylated recombinant human hyaluronidase PH20
PKs: pharmacokinetics
PR: partial response
RT: radiation therapy
SD: stable disease
TAMs: tumor-associated macrophages
TILs: tumor-infiltrating lymphocytes
TMB: tumor mutation burden
TME: tumor microenvironment
TNF: tumor necrosis factor alpha
Tregs: regulatory T-cells

## References

[1] Gong J, Chehrazi-Raffle A, Reddi S, Salgia R (2018). Development of PD-1 and PD-L1 inhibitors as a form of cancer immunotherapy: a comprehensive review of registration trials and future considerations. J Immunother Cancer.

[2] Geng L, Huang D, Liu J, Qian Y, Deng J, Li D (2008). B7-H1 up-regulated expression in human pancreatic carcinoma tissue associates with tumor progression. J Cancer Res Clin Oncol.

[3] Loos M, Giese NA, Kleeff J, Giese T, Gaida MM, Bergmann F (2008). Clinical significance and regulation of the costimulatory molecule B7-H1 in pancreatic cancer. Cancer Lett.

[4] Nomi T, Sho M, Akahori T, Hamada K, Kubo A, Kanehiro H (2007). Clinical significance and therapeutic potential of the programmed death-1 ligand/programmed death-1 pathway in human pancreatic cancer. Clin Cancer Res.

[5] Okudaira K, Hokari R, Tsuzuki Y, Okada Y, Komoto S, Watanabe C (2009). Blockade of B7-H1 or B7-DC induces an anti-tumor effect in a mouse pancreatic cancer model. Int J Oncol.

[6] Brahmer JR, Tykodi SS, Chow LQ, Hwu WJ, Topalian SL, Hwu P (2012). Safety and activity of anti-PD-L1 antibody in patients with advanced cancer. N Engl J Med.

[7] Herbst RS, Soria JC, Kowanetz M, Fine GD, Hamid O, Gordon MS (2014). Predictive correlates of response to the anti-PD-L1 antibody MPDL3280A in cancer patients. Nature.

[8] Patnaik A, Kang SP, Rasco D, Papadopoulos KP, Elassaiss-Schaap J, Beeram M (2015). Phase I study of pembrolizumab (MK-3475; anti-PD-1 monoclonal antibody) in patients with advanced solid tumors. Clin Cancer Res.

[9] Royal RE, Levy C, Turner K, Mathur A, Hughes M, Kammula US (2010). Phase 2 trial of single agent Ipilimumab (anti-CTLA-4) for locally advanced or metastatic pancreatic adenocarcinoma. J Immunother.

[10] Jenkins RW, Barbie DA, Flaherty KT (2018). Mechanisms of resistance to immune checkpoint inhibitors. Br J Cancer.

[11] Foley K, Kim V, Jaffee E, Zheng L (2016). Current progress in immunotherapy for pancreatic cancer. Cancer Lett.

[12] Knudsen ES, Vail P, Balaji U, Ngo H, Botros IW, Makarov V (2017). Stratification of pancreatic ductal adenocarcinoma: combinatorial genetic, stromal, and immunologic markers. Clin Cancer Res.

[13] Sahin IH, Askan G, Hu ZI, O’Reilly EM (2017). Immunotherapy in pancreatic ductal adenocarcinoma: an emerging entity?. Ann Oncol.

[14] Sharma P, Hu-Lieskovan S, Wargo JA, Ribas A (2017). Primary, adaptive, and acquired resistance to cancer immunotherapy. Cell.

[15] Brooks J, Fleischmann-Mundt B, Woller N, Niemann J, Ribback S, Peters K (2018). Perioperative, spatiotemporally coordinated activation of T and NK cells prevents recurrence of pancreatic cancer. Cancer Res.

[16] Bozeman EN, Gao N, Qian W, Wang A, Yang L (2015). Synergistic effect of targeted chemotherapy delivery using theranostic nanoparticles and PD-L1 blockade in an orthotopic mouse pancreatic cancer model [abstract]. Cancer Immunol Res.

[17] Zhu Y, Knolhoff BL, Meyer MA, Nywening TM, West BL, Luo J (2014). CSF1/CSF1R blockade reprograms tumor-infiltrating macrophages and improves response to T-cell checkpoint immunotherapy in pancreatic cancer models. Cancer Res.

[18] Jiang H, Hegde S, Knolhoff BL, Zhu Y, Herndon JM, Meyer MA (2016). Targeting focal adhesion kinase renders pancreatic cancers responsive to checkpoint immunotherapy. Nat Med.

[19] Lu C, Paschall AV, Shi H, Savage N, Waller JL, Sabbatini ME (2017). The MLL1-H3K4me3 axis-mediated PD-L1 expression and pancreatic cancer immune evasion. J Natl Cancer Inst.

[20] Iorio V, Rosati A, D’Auria R, De Marco M, Marzullo L, Basile A (2018). Combined effect of anti-BAG3 and anti-PD-1 treatment on macrophage infiltrate, CD8+ T cell number and tumour growth in pancreatic cancer. Gut.

[21] Lu C, Talukder A, Savage NM, Singh N, Liu K (2017). JAK-STAT-mediated chronic inflammation impairs cytotoxic T lymphocyte activation to decrease anti-PD-1 immunotherapy efficacy in pancreatic cancer. Oncoimmunology.

[22] Zhang Y, Velez-Delgado A, Mathew E, Li D, Mendez FM, Flannagan K (2017). Myeloid cells are required for PD-1/PD-L1 checkpoint activation and the establishment of an immunosuppressive environment in pancreatic cancer. Gut.

[23] Smith JP, Wang S, Nadella S, Jablonski SA, Weiner LM (2018). Cholecystokinin receptor antagonist alters pancreatic cancer microenvironment and increases efficacy of immune checkpoint antibody therapy in mice. Cancer Immunol Immunother.

[24] Gonda TA, Tycko B, Salas MC, Do C, Fang J, Olive KP (2017). Combination therapy with a hypomethylating drug (decitabine) plus an immune checkpoint inhibitor (anti-PD-1H) in the KPC mouse model of pancreatic cancer [abstract]. Gastroenterology.

[25] Pilon-Thomas S, Kodumudi KN, El-Kenawi AE, Russell S, Weber AM, Luddy K (2016). Neutralization of tumor acidity improves antitumor responses to immunotherapy. Cancer Res.

[26] Ibrahim-Hashim AA, Abrahams D, Xu L, Centeno B, Sunassee E, Abddelgader R (2017). Targeting tumor acidity with the LDHA inhibitor (FX11) and CAIX inhibitor (DH348) overcomes resistance to PD-1 blockade and inhibits metastasis in a pancreatic cancer model [abstract]. Cancer Res.

[27] Rosengren S, Clift R, Zimmerman SJ, Souratha J, Thompson BJ, Blouw B (2016). PEGylated recombinant hyaluronidase PH20 (PEGPH20) enhances checkpoint inhibitor efficacy in syngeneic mouse models of cancer [abstract]. Cancer Res.

[28] Soares KC, Rucki AA, Wu AA, Olino K, Xiao Q, Chai Y (2015). PD-1/PD-L1 blockade together with vaccine therapy facilitates effector T-cell infiltration into pancreatic tumors. J Immunother.

[29] Kim V, Foley K, Soares K, Rucki A, Lauer P, Brockstedt D (2016). Sequential treatment with a listeria-based vaccine and PD-1 blockade antibody improves survival in a murine model of pancreatic ductal adenocarcinoma [abstract]. HPB.

[30] Zheng W, Skowron KB, Namm JP, Burnette B, Fernandez C, Arina A (2016). Combination of radiotherapy and vaccination overcomes checkpoint blockade resistance. Oncotarget.

[31] Joyce JA, Fearon DT (2015). T cell exclusion, immune privilege, and the tumor microenvironment. Science.

[32] Feig C, Jones JO, Kraman M, Wells RJ, Deonarine A, Chan DS (2013). Targeting CXCL12 from FAP-expressing carcinoma-associated fibroblasts synergizes with anti-PD-L1 immunotherapy in pancreatic cancer. Proc Natl Acad Sci U S A.

[33] Miao L, Li J, Liu Q, Feng R, Das M, Lin CM (2017). Transient and local expression of chemokine and immune checkpoint traps to treat pancreatic cancer. ACS Nano.

[34] Mace TA, Shakya R, Pitarresi JR, Swanson B, McQuinn CW, Loftus S (2018). IL-6 and PD-L1 antibody blockade combination therapy reduces tumour progression in murine models of pancreatic cancer. Gut.

[35] Zhao Y, Shen M, Feng Y, He R, Xu X, Xie Y (2017). Regulatory B cells induced by pancreatic cancer cell-derived interleukin-18 promote immune tolerance via the PD-1/PD-L1 pathway. Oncotarget.

[36] Steele CW, Karim SA, Leach JDG, Bailey P, Upstill-Goddard R, Rishi L (2016). CXCR2 inhibition profoundly suppresses metastases and augments immunotherapy in pancreatic ductal adenocarcinoma. Cancer Cell.

[37] Janson C, Jung H, Ertl L, Liu S, Dang T, Zeng Y (2017). Inhibition of CCR2 potentiates checkpoint inhibitor immunotherapy in murine model of pancreatic cancer [abstract]. Cancer Res.

[38] Dougan M, Ingram JR, Jeong HJ, Mosaheb MM, Bruck PT, Ali L (2018). Targeting cytokine therapy to the pancreatic tumor microenvironment using PD-L1-specific VHHs. Cancer Immunol Res.

[39] Kobold S, Grassmann S, Chaloupka M, Lampert C, Wenk S, Kraus F (2015). Impact of a new fusion receptor on PD-1-mediated immunosuppression in adoptive T cell therapy. J Natl Cancer Inst.

[40] Rataj F, Kraus F, Grassmann S, Chaloupka M, Ogonek J, Zhang J (2018). Preclinical characterization of a PD-1-CD28 fusion receptor in CD4+ T cells for T cell-based immunotherapy of pancreatic cancer and Non-Hodgkin Lymphoma [abstract]. Eur J Cancer.

[41] Luheshi NM, Coates-Ulrichsen J, Harper J, Mullins S, Sulikowski MG, Martin P (2016). Transformation of the tumour microenvironment by a CD40 agonist antibody correlates with improved responses to PD-L1 blockade in a mouse orthotopic pancreatic tumour model. Oncotarget.

[42] Winograd R, Byrne KT, Evans RA, Odorizzi PM, Meyer AR, Bajor DL (2015). Induction of T-cell immunity overcomes complete resistance to PD-1 and CTLA-4 blockade and improves survival in pancreatic carcinoma. Cancer Immunol Res.

[43] Mehla K, Tremayne J, Grunkemeyer JA, O’Connell KA, Steele MM, Caffrey TC (2018). Combination of mAb-AR20.5, anti-PD-L1 and PolyICLC inhibits tumor progression and prolongs survival of MUC1.Tg mice challenged with pancreatic tumors. Cancer Immunol Immunother.

[44] Mihailidou C, Papakotoulas P, Schizas D, Papalampros A, Vailas M, Felekouras E (2017). Effect of ciclopirox olamine in immunotherapy effect by stimulating immunogenic cell death in pancreatic cancer [abstract]. Ann Oncol.

[45] Le DT, Lutz E, Uram JN, Sugar EA, Onners B, Solt S (2013). Evaluation of ipilimumab in combination with allogeneic pancreatic tumor cells transfected with a GM-CSF gene in previously treated pancreatic cancer. J Immunother.

[46] Nesselhut J, Marx D, Cillien N, Lange H, Regalo G, Herrmann M (2015). Dendritic cells generated with PDL-1 checkpoint blockade for treatment of advanced pancreatic cancer [abstract]. J Clin Oncol.

[47] Nesselhut J, Marx D, Lange H, Regalo G, Cillien N, Chang RY (2016). Systemic treatment with anti-PD-1 antibody nivolumab in combination with vaccine therapy in advanced pancreatic cancer [abstract]. J Clin Oncol.

[48] Aglietta M, Barone C, Sawyer MB, Moore MJ, Miller WH, Bagalà C (2014). A phase I dose escalation trial of tremelimumab (CP-675,206) in combination with gemcitabine in chemotherapy-naive patients with metastatic pancreatic cancer. Ann Oncol.

[49] Weiss GJ, Waypa J, Blaydorn L, Coats J, McGahey K, Sangal A (2017). A phase Ib study of pembrolizumab plus chemotherapy in patients with advanced cancer (PembroPlus). Br J Cancer.

[50] Weiss GJ, Blaydorn L, Beck J, Bornemann-Kolatzki K, Urnovitz H, Schütz E (2018). Phase Ib/II study of gemcitabine, nab-paclitaxel, and pembrolizumab in metastatic pancreatic adenocarcinoma. Invest New Drugs.

[51] Kalyan A, Kircher SM, Mohindra NA, Nimeiri HS, Maurer V, Rademaker A (2016). Ipilimumab and gemcitabine for advanced pancreas cancer: a phase Ib study [abstract]. J Clin Oncol.

[52] Wainberg ZA, Hochster HS, George B, Gutierrez M, Johns ME, Chiorean EG (2017). Phase I study of nivolumab (nivo) + nab-paclitaxel (nab-P) ± gemcitabine (Gem) in solid tumors: Interim results from the pancreatic cancer (PC) cohorts [abstract]. J Clin Oncol.

[53] Katz MHG, Varadhachary GR, Bauer TW, Acquavella N, Merchant NB, Le TM (2017). Preliminary safety data from a randomized multicenter phase Ib/II study of neoadjuvant chemoradiation therapy (CRT) alone or in combination with pembrolizumab in patients with resectable or borderline resectable pancreatic cancer [abstract]. J Clin Oncol.

[54] Renouf DJ, Dhani NC, Kavan P, Jonker DJ, Chia-chi Wei A, Hsu T (2018). The Canadian Cancer Trials Group PA.7 trial: results from the safety run in of a randomized phase II study of gemcitabine (GEM) and nab-paclitaxel (Nab-P) versus GEM, nab-P, durvalumab (D), and tremelimumab (T) as first-line therapy in metastatic pancreatic ductal adenocarcinoma (mPDAC) [abstract]. J Clin Oncol.

[55] Friedlander M, Meniawy T, Markman B, Mileshkin LR, Harnett PR, Millward M (2017). A phase 1b study of the anti-PD-1 monoclonal antibody BGB-A317 (A317) in combination with the PARP inhibitor BGB-290 (290) in advanced solid tumors [abstract]. J Clin Oncol.

[56] Fujiwara Y, Shitara K, Shimizu T, Yonemori K, Matsubara N, Ohno I (2018). INCB024360 (Epacadostat) monotherapy and in combination with pembrolizumab in patients with advanced solid tumors: primary results from first-in-Japanese phase I study (KEYNOTE-434) [abstract]. Mol Cancer Ther.

[57] O’Reilly EM, Oh D, Dhani N, Renouf DJ, Lee MA, Sun W (2018). A randomized phase 2 study of durvalumab monotherapy and in combination with tremelimumab in patients with metastatic pancreatic ductal adenocarcinoma (mPDAC): ALPS study [abstract]. J Clin Oncol.

[58] Strauss J, Heery CR, Schlom J, Madan RA, Cao L, Kang Z (2018). Phase I trial of M7824 (MSB0011359C), a bifunctional fusion protein targeting PD-L1 and TGFβ, in advanced solid tumors. Clin Cancer Res.

[59] Banerjee K, Kumar S, Ross KA, Gautam S, Poelaert B, Nasser MW (2018). Emerging trends in the immunotherapy of pancreatic cancer. Cancer Lett.

[60] Xu JW, Wang L, Cheng YG, Zhang GY, Hu SY, Zhou B (2018). Immunotherapy for pancreatic cancer: a long and hopeful journey. Cancer Lett.

[61] Feng M, Xiong G, Cao Z, Yang G, Zheng S, Song X (2017). PD-1/PD-L1 and immunotherapy for pancreatic cancer. Cancer Lett.

[62] Chowdhury PS, Chamoto K, Honjo T (2018). Combination therapy strategies for improving PD-1 blockade efficacy: a new era in cancer immunotherapy. J Intern Med.

[63] Mahoney KM, Rennert PD, Freeman GJ (2015). Combination cancer immunotherapy and new immunomodulatory targets. Nat Rev Drug Discov.

[64] Sharma P, Allison JP (2015). Immune checkpoint targeting in cancer therapy: toward combination strategies with curative potential. Cell.

[65] Thomas-Schoemann A, Lemare F, Mongaret C, Bermudez E, Chéreau C, Nicco C (2011). Bystander effect of vinorelbine alters antitumor immune response. Int J Cancer.

[66] Smyth MJ, Ngiow SF, Ribas A, Teng MW (2016). Combination cancer immunotherapies tailored to the tumour microenvironment. Nat Rev Clin Oncol.

[67] Sherman MH, Yu RT, Engle DD, Ding N, Atkins AR, Tiriac H (2014). Vitamin D receptor-mediated stromal reprogramming suppresses pancreatitis and enhances pancreatic cancer therapy. Cell.

[68] Vonderheide RH (2018). The immune revolution: a case for priming, not checkpoint. Cancer Cell.

[69] Ott PA, Hodi FS, Kaufman HL, Wigginton JM, Wolchok JD (2017). Combination immunotherapy: a road map. J Immunother Cancer.

[70] Gong J, Le TQ, Massarelli E, Hendifar AE, Tuli R (2018). Radiation therapy and PD-1/PD-L1 blockade: the clinical development of an evolving anticancer combination. J Immunother Cancer.

[71] Antonia SJ, Villegas A, Daniel D, Vicente D, Murakami S, Hui R (2017). Durvalumab after chemoradiotherapy in stage III non-small-cell lung cancer. N Engl J Med.

[72] Forde PM, Chaft JE, Smith KN, Anagnostou V, Cottrell TR, Hellmann MD (2018). Neoadjuvant PD-1 blockade in resectable lung cancer. N Engl J Med.

[73] Weber J, Mandala M, Del Vecchio M, Gogas HJ, Arance AM, Cowey CL (2017). Adjuvant nivolumab versus ipilimumab in resected stage III or IV melanoma. N Engl J Med.

[74] Eggermont AM, Chiarion-Sileni V, Grob JJ, Dummer R, Wolchok JD, Schmidt H (2015). Adjuvant ipilimumab versus placebo after complete resection of high-risk stage III melanoma (EORTC 18071): a randomised, double-blind, phase 3 trial. Lancet Oncol.

[75] Eggermont AMM, Blank CU, Mandala M, Long GV, Atkinson V, Dalle S (2018). Adjuvant pembrolizumab versus placebo in resected stage III melanoma. N Engl J Med.

[76] Gao HL, Liu L, Qi ZH, Xu HX, Wang WQ, Wu CT (2018). The clinicopathological and prognostic significance of PD-L1 expression in pancreatic cancer: a meta-analysis. Hepatobiliary Pancreat Dis Int.

[77] Zhuan-Sun Y, Huang F, Feng M, Zhao X, Chen W, Zhu Z (2017). Prognostic value of PD-L1 overexpression for pancreatic cancer: evidence from a meta-analysis. Onco Targets Ther.

[78] Campbell BB, Light N, Fabrizio D, Zatzman M, Fuligni F, de Borja R (2017). Comprehensive analysis of hypermutation in human cancer. Cell.

[79] Hu ZI, Shia J, Stadler ZK, Varghese AM, Capanu M, Salo-Mullen E (2018). Evaluating mismatch repair deficiency in pancreatic adenocarcinoma: challenges and recommendations. Clin Cancer Res.

[80] Balli D, Rech AJ, Stanger BZ, Vonderheide RH (2017). Immune cytolytic activity stratifies molecular subsets of human pancreatic cancer. Clin Cancer Res.

[81] Pushalkar S, Hundeyin M, Daley D, Zambirinis CP, Kurz E, Mishra A (2018). The pancreatic cancer microbiome promotes oncogenesis by induction of innate and adaptive immune suppression. Cancer Discov.

[82] Zhang J, Wolfgang CL, Zheng L (2018). Precision immuno-oncology: prospects of individualized immunotherapy for pancreatic cancer. Cancers (Basel).

